# Regeneration Capacity of Small Clonal Fragments of the Invasive *Mikania micrantha* H.B.K.: Effects of Burial Depth and Stolon Internode Length

**DOI:** 10.1371/journal.pone.0084657

**Published:** 2013-12-18

**Authors:** Xiaoxia Li, Yide Shen, Qiaoqiao Huang, Zhiwei Fan, Dongdong Huang

**Affiliations:** Environment and Plant Protection Institute, Chinese Academy of Tropical Agricultural Sciences, Haikou, China; Beijing Forestry University, China

## Abstract

The perennial stoloniferous herbaceous vine *Mikania micrantha* H.B.K. is among the most noxious exotic invaders in China and the world. Disturbance can fragment stolons of *M. micrantha* and disperse these fragments over long distances or bury them in soils at different depths. To test their regeneration capacity, single-node stolon fragments with stolon internode lengths of 0, 3, 6 and 12 cm were buried in soil at 0, 2, 5 and 8 cm depths, respectively. The fragments were growing for nine weeks, and their emergence status, growth and morphological traits were measured. The results indicated that increasing burial depth significantly decreased survival rate and increased the emergence time of the *M. micrantha* plants. At an 8-cm burial depth, very few fragments (2.19%) emerged and survived. Burial did not affect the total biomass and root to shoot ratio of the surviving *M. micrantha* plants that emerged from the 0- and 2-cm burial depths. Increasing internode length significantly increased survival rate and growth measures, but there was no interaction effect with burial depth for any traits measured. These results suggest that *M. micrantha* can regenerate from buried stolon fragments, and thus, disturbance may contribute to the spread of this exotic invader. Any human activities producing stolon fragments or facilitating dispersal should be avoided.

## Introduction

Understanding the mechanisms explaining the establishment and spread of exotic species is a leading topic in invasion biology and applied ecology. Successful invasion depends both on the attributes of the invader and the characteristics of the recipient community [1]. The establishment and spread of plant invaders are generally facilitated by community disturbance [2]. Disturbance can mediate competitive interactions between exotic invaders and natives in ways that often favor the exotics [3]. In addition, disturbance often leads to increased resource supply into a community or decreased resource uptake by resident plants, which provides niche opportunities for the establishment and proliferation of exotic plants [4]. The overall effect of disturbance on invasion depends also on species attributes. Life-history traits, such as regrowth capacity and vegetative propagation, may significantly determine the potential for a clonal plant invader to benefit from disturbances [5,6]. 

Many noxious plant invaders are clonals [7]. The importance of spread and regeneration by clonal fragments has been demonstrated in many clonal invaders [7-10], with some spreading almost solely by vegetative reproduction [11,12]. The generation and spread of clonal fragments can be caused by disturbances such as flooding, typhoons, landslips, animal trampling and human activities. These disturbances can also destabilize soil substrates, thus causing clonal fragments of various sizes to be buried in soils at different depths [5,6]. The potential for regeneration by clonal fragments after disturbance may depend on the combination of habitat conditions (burial depth) and biological properties (fragment size and resprouting ability). A few studies have tested the effects of burial on the resprouting ability of fragmented clonal invaders, but more studies have focused on fragmented roots [6,9,13,14] and not stolons [15]. A previous study examined the effects of burial on the regeneration capacity of fragmented stolons of another noxious invader in China, *Alternanthera philoxeroides* [15], but the generalizability of the results remains to be tested, especially considering that there are many stoloniferous clonal invaders in China, such as *Mikania micrantha*, *Ipomoea cairica* and *Wedelia trilobata* [7]. It is also important to obtain a better understanding of the regeneration capacity of fragmented stolons because compared with fragmented roots, fragmented stolons have a higher probability to be produced and carried away long distances, which significantly contributes to invasion spread [16].

In this study, the regeneration capacity of stolon fragments from the invasive *M. micrantha* was assessed. *M. micrantha* is one of the top 10 worst weeds in the world and is now rapidly spreading in South China [17]. *M. micrantha* poses serious threats to forests, farmlands and orchards [17]. Research continues to obtain more information on this species, including on its distribution [17], biology and ecology [17], habitat infested and impacts [18-20], population genetics [21] and control [22]. However, information is lacking on the regeneration capacity of its clonal fragments. As an island and also a province with intense agricultural activities, disturbances such as flooding, typhoons, landslips, animal trampling and farming activities are particularly common in Hainan province of China, where *M. micrantha* is spreading, and the experiment was conducted. These disturbances can generate and spread clonal fragments of *M. micrantha*.

To assess stolon regeneration, we conducted a greenhouse experiment with stolon fragments of various sizes buried at varying soil depths. Specifically, we address the following two hypotheses: (1) stolon fragments of *M. micrantha* have a high regeneration capacity, and (2) the regeneration capacity and subsequent growth of stolon fragments is related to both fragment size and burial depth. 

## Materials and Methods

### Ethics statement

 The site where we sampled plant materials of *M. micrantha* did not belong to any farms, national parks or protected areas, so no permissions were required for collecting plant samples. The site also did not contain any endangered or protected species. 

### The species

*Mikania micrantha* H.B.K. is a stoloniferous perennial herbaceous vine of the Asteraceae family that originates from tropical Central and South America [17]. It was introduced into China in 1919 [17]. It has spread extensively in Guangdong province and has invaded many disturbed forests, farmlands, wastelands, roadsides and orchards [23]. *M. micrantha* was also discovered in Hainan province in 2003 and has spread to more than ten counties or cities since then [24]. *M. micrantha* spreads and establishes populations through both seeds and clonal fragments. Once established, *M. micrantha* has the potential to cover the whole habitat through stolon elongation and ramification. The habitats *M. micrantha* has invaded are subject to frequent disturbances. Our field observations suggest that disturbances such as flooding, typhoons, landslips, animal trampling and human activities (including incomplete manual or mechanical control) can break plant clones of *M. micrantha* into small pieces, including single-node fragments, and bury these fragments in soils at varying depths. Such small clonal fragments may regenerate in the disturbance location or establish new populations if they are carried away to other places. 

### Material preparation and experimental design

For the experiment, stolon fragments of *M. micrantha* were collected in an artificial plantation along a highroad in the suburbs of Haikou in Hainan province, China. On June 3, 2013, sufficient stolon fragments for the experiment were collected and taken into the laboratory. A sample of stolons of similar thickness was chosen for fragmentation. The stolons were cut into single-node ramets with varying internode length, and roots and leaves were removed to avoid the potential effects of their presence [16]. A total of 1280 stolon fragments were used for the experiment, and another 60 were used for initial measurement. The diameter of the stolon fragments was 2.47±0.04 mm (mean ± SE, *N* = 45), and the dry weights were 28.5±3.8, 52.6±4.5, 102.9±7.6 and 184.6±14.1 mg (mean ± SE, *N* = 15) for the 0-, 3-, 6- and 12-cm-long fragments, respectively. 

A split-plot designed experiment was conducted with burial depth as a whole plot factor and internode length as a subplot factor. The experiment consisted of four treatments of burial depth, i.e., 0, 2, 5 and 8 cm, combined with four treatments of stolon internode length, i.e., 0, 3, 6 and 12 cm. In the field, stolon fragments may be buried in soils deeper than 8 cm, but in this study, 8 cm was used as the maximum burial depth. For the fragments buried at 0 cm, stolons were placed directly on the soil. Each fragment consisted of both proximal (before and thus developmentally older than the node) and distal internodes (after and thus developmentally younger than the node), each having half of the total length. For instance, for the 12-cm internode length treatment, both the proximal and distal internodes of the fragment were cut into 6-cm long lengths. A total of 32 plastic containers (60 cm × 34 cm × 16 cm deep) were each filled with a 10-cm deep mixture of sand, peat and soil with a volume ratio of 1:1:1. Each container was further divided into four equal boxes (30 cm × 17 cm × 16 cm deep) with each randomly assigned to one of the four internode length treatments, and in each box, ten fragments with the same internode length were placed horizontally in ten evenly spaced positions. Therefore, in each container there were 40 fragments, consisting of ten fragments of each of the four internode length types (0, 3, 6 and 12 cm). Fragments within a container were buried at the same depth. There were eight replicates of containers for each of the four burial depth treatments (0, 2, 5 and 8 cm). Tap water was supplied each day to keep the soil moist. Woody frames were provided for emerged plants to climb. During the experiment, the positions of the containers were randomly changed once a week. 

The experiment was conducted in a greenhouse at Chinese Academy of Tropical Agricultural Sciences, Danzhou. Because one common habitat *M. micrantha* invades is disturbed forests and plantations, the containers were placed under a shade cloth to simulate light conditions in forest understory, and the maximum photosynthetically active radiation at noon in sunny days was approximately 300 micromoles m^−2^ s^−1^ (approximately 20% of that under full sun). 

### Measurements

 All clonal fragments were planted on June 4, 2013. Plants were growing for nine weeks and harvested on August 6, 2013. During the experiment, plant emergence was recorded every day. A plant was coded as ‘‘emerged’’ if the above-ground part of new shoots sprouting from the original fragment exceeded 1 cm in length. At harvest, we recorded the number of surviving plants in each box and measured plant shoot height. Then, each plant was divided into leaves, stolons, roots and the original fragments, before being oven-dried at 70°C for 48 h and weighed. 

### Data analyses

 We calculated the emergence rate and survival rate in each box. The mean values of emergence time (days since planting), total biomass (the sum of leaf, stolon and root biomass), leaf biomass, stolon biomass, root biomass, root to shoot ratio (root biomass/shoot biomass; shoot biomass is the sum of leaf biomass and stolon biomass) and shoot height of the surviving plants in each box were also calculated. These derived data were used for the following analyses. 

 Split-plot ANOVAs with containers (i.e., burial depth) as the whole plots and boxes (i.e., internode length) as the subplots were used to determine the effects of burial depth, internode length and their interactions on the emergence rate, emergence time, survival rate and growth and morphological traits of *M. micrantha* [25]. The significance of burial depth was tested against the whole plot error, and the significance of internode length and its interaction with burial depth was tested against the subplot error. If necessary, data were ln-transformed prior to analysis. Burial depth and internode length were considered as fixed effects, and type III sum of squares was used to determine *F*-values. 

 It was not possible to perform these analyses for plants buried at 5 and 8 cm depths because of high mortality. Thus, we only performed these analyses for plants buried at 0 and 2 cm. However, as data from plants with 6- and 12-cm internode length that were buried at 5 cm depth can be analyzed, additional one-way ANOVAs with post-hoc Duncan’s tests (with *P*=0.05) were conducted to examine the differences of means among the ten treatments (0- and 2-cm burial depth treatments with each combined with four internode length treatments, 5-cm burial depth with 6-cm internode length treatment and 5-cm burial depth with 12-cm internode length treatment). The results from one-way ANOVAs are presented in the figures. Data from the plants buried at 8 cm were not analyzed because of the low sample size. Boxes that had no surviving plants were excluded for analyses of emergence time and growth and morphological measures. All analyses were conducted with the SPSS statistical program, version 16.0 (SPSS, Chicago, IL, USA). 

## Results

### Emergence rate, emergence time and survival rate

**Figure 1:**
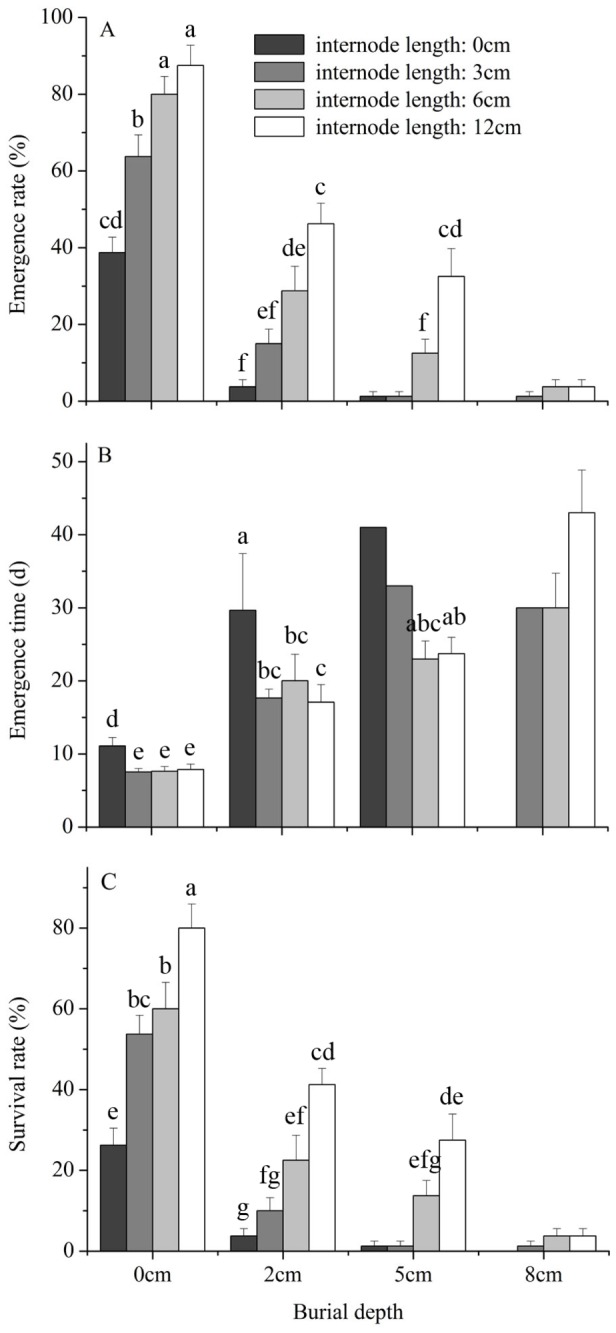
Effects of burial depth and stolon internode length on emergence rate, emergence time and survival rate of *Mikania micrantha*. Emergence time is the number of days between planting and emergence. Error bars represent the mean ± SE. One-way ANOVAs with post-hoc Duncan’s tests were used for the multiple comparison analyses (data on emergence time were ln-transformed prior to analyses), and significant differences (at the significance level of *P*=0.05) between two treatments are marked with the use of different symbols.

 For fragments buried at 0 and 2 cm, increasing burial depth significantly decreased emergence and survival rate and increased emergence time, and increasing internode length increased emergence and survival rate and decreased emergence time (Figure 1, Table 1). Differences in emergence time among internode length treatments were primarily driven by the long emergence time of fragments with a 0-cm internode length (Figure 1B). At 5-cm burial depth, only two out of 160 fragments with 0- and 3-cm internode length emerged and survived, and fragments with 6-cm and 12-cm internode length differed in emergence rate but not in survival rate and emergence time (Figure 1). Only seven (one with 3-, three with 6-, and three with 12-cm internode length) out of 320 plants (2.19%) buried at 8 cm emerged and survived. 

**Table 1:** Split-plot ANOVAs for effects of burial depth, stolon internode length and the interaction on emergence rate, emergence time and survival rate of *Mikania micrantha*.

Effect	Burial (B)			Length (L)			B×L	
	DF	*F*		DF	*F*		DF	*F*
Emergence rate	1,14	158.3***		3,42	34.5***		3,42	1.2^ns^
Emergence time^a^	1,14	119.4***		3,34	5.3**		3,34	0.5^ns^
Survival rate	1,14	154.7***		3,42	28.4***		3,42	1.6^ns^

Degree of freedom (DF), *F* values and the significance levels (*** *P*<0.001, ** *P*<0.01 and ns *P*>0.05) are given.

^a^ Data were ln-transformed prior to analysis.

### Biomass

**Figure 2:**
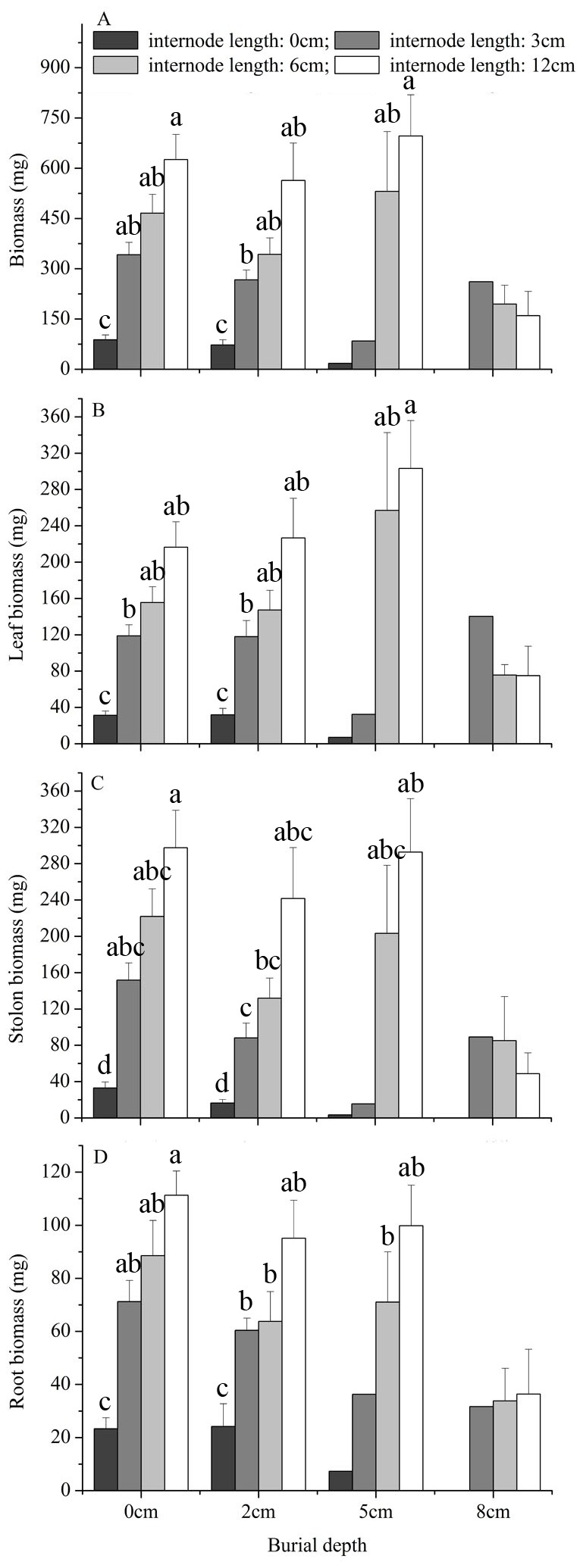
Effects of burial depth and stolon internode length on growth of *Mikania micrantha*. Total biomass, leaf biomass, stolon biomass and root biomass of the surviving fragments are given. Error bars represent the mean ± SE. One-way ANOVAs with post-hoc Duncan’s tests were used for the multiple comparison analyses (data were ln-transformed prior to analyses), and significant differences (at the significance level of *P*=0.05) between two treatments are marked with the use of different symbols.

For fragments buried at 0 and 2 cm, increasing burial depth did not change total biomass, leaf biomass and root biomass but decreased the stolon biomass of the surviving *M. micrantha* plants (Figure 2, Table 2). Increasing internode length increased total biomass, leaf biomass, shoot biomass and root biomass of the surviving *M. micrantha* plants (Figure 2, Table 2). For fragments with a 6-cm internode length, compared with fragments buried at 0- and 2-cm depths, burial at 5-cm depth also did not decrease total biomass, leaf biomass, stolon biomass and root biomass of the surviving *M. micrantha* plants (Figure 2). Similar results were also found for fragments with a 12-cm internode length (Figure 2). 

**Table 2:** Split-plot ANOVAs for effects of burial depth, stolon internode length and the interaction on growth and morphological characteristics of *Mikania micrantha*.

Effect	Burial (B)			Length (L)			B×L	
	DF	*F*		DF	*F*		DF	*F*
Total biomass^a^	1,14	1.8^ns^		3,34	39.3***		3,34	0.1^ns^
Leaf biomass^a^	1,14	0.1^ns^		3,34	38.2***		3,34	0.1^ns^
Stolon biomass^a^	1,14	5.9*		3,34	28.8***		3,34	0.3^ns^
Root biomass^a^	1,14	1.1^ns^		3,34	35.5***		3,34	0.4^ns^
Shoot height	1,14	5.5*		3,34	17.4***		3,34	0.0^ns^
Root to shoot ratio^b^	1,14	0.5^ns^		3,34	8.9***		3,34	0.5^ns^

Degree of freedom (DF), *F* values and the significance levels (*** *P*<0.001, * *P*<0.05 and ns *P*>0.05) are given.

^a^ Data were ln-transformed prior to analysis.

^b^ Data were first multiplied by 10 and then ln-transformed prior to analysis.

### Root to shoot ratio and shoot height

**Figure 3:**
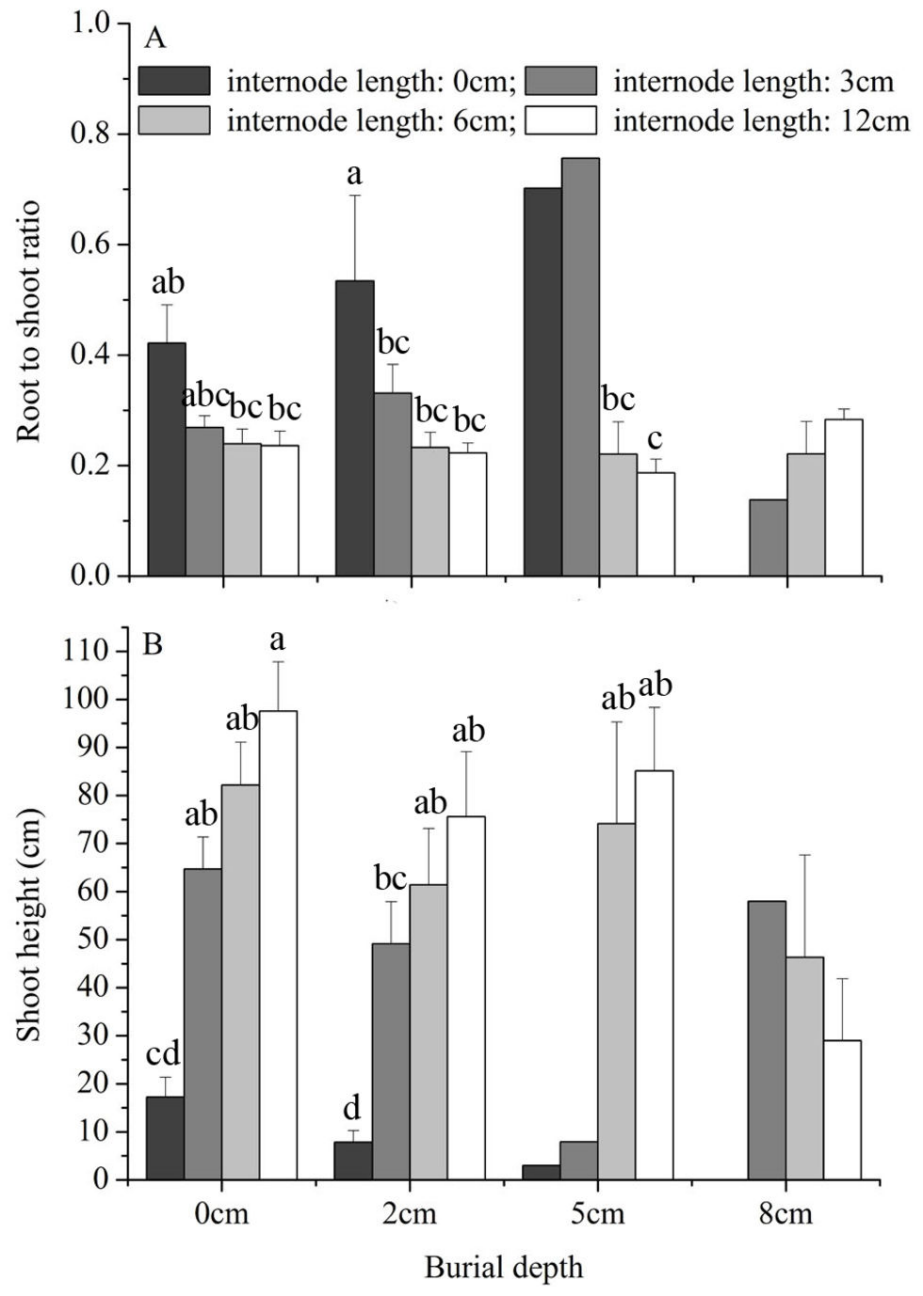
Effects of burial depth and stolon internode length on morphology of *Mikania micrantha*. Root to shoot ratio and shoot height are given. Error bars represent the mean ± SE. One-way ANOVAs with post-hoc Duncan’s tests were used for the multiple comparison analyses (data on root to shoot ratio were first multiplied by 10 and then ln-transformed prior to analyses), and significant differences (at the significance level of *P*=0.05) between two treatments are marked with the use of different symbols.

 For fragments buried at 0 and 2 cm, increasing burial depth did not affect root to shoot ratio of the surviving *M. micrantha* plants, but increasing internode length decreased the root to shoot ratio of the surviving *M. micrantha* plants (Figure 3A, Table 2). The root to shoot ratio differences among internode length treatments were primarily driven by the high root to shoot ratio of fragments with a 0-cm internode length (Figure 3A). For plants with an internode length of either 6 cm or 12 cm, compared with fragments buried at 0 cm and 2 cm, burial at 5 cm also did not affect root to shoot ratio of the surviving *M. micrantha* plants (Figure 3A). 

 For fragments buried at 0 and 2 cm, the shoot height decreased with burial depth but increased with internode length (Figure 3B, Table 2). For plants with an internode length of either 6 cm or 12 cm, compared with burial at 0 cm and 2 cm, burial at 5 cm did not affect the shoot height of the surviving *M. micrantha* plants (Figure 3B). 

## Discussion

Spread and regeneration by clonal fragments is common in exotic invasive plants and may play a significant role in their population dynamics [7]. The exotic invader investigated here could regenerate from stolon fragments, and the regeneration capacity and subsequent growth depended on the fragment size and burial depth. 

 Emergence and survival of *M. micrantha* stolon fragments were negatively associated with burial depth and positively associated with fragment size, while emergence time was positively associated with burial depth and negatively associated with fragment size. At a 0-cm burial depth, the emergence rate (67.5%) and survival rate (55%) were high. The emergence rate almost reached zero for fragments that were buried at 8 cm (2.19%) and for fragments with a 0- and 3-cm internode length that were buried at 5 cm (1.25%). Burial has been assumed to be a major environmental stress reducing plant performance [26,27], and many other studies also have indicated that burial significantly decreased the emergence and survival of rhizome and stolon fragments [5,13,15,28-30]. Compared with another stoloniferous noxious invader in China, *A. philoxeroides*, for which fragment survival decreased from 60.9% at a 0-cm burial depth to 16.9% at an 8-cm burial depth [15], the survival of the *M. micrantha* fragments was more sensitive to burial (i.e., only 2.19% at an 8-cm burial depth). Interspecies differences in regeneration capacity have also been reported in other studies [13,16,31,32] and may be attributed to differences in vigor or the amount of reserves stored, as well as in factors other than species traits, e.g., soil type [33], water content [10], light conditions [34] and temperature. These differences in regeneration capacity after burial may partly explain species differences in invasiveness. On the other hand, the finding that increasing internode length increased the emergence and survival rate is consistent with previous findings [13,15,35,36] and suggests that reserves stored in stolon internodes can be remobilized and reused for regeneration of clonal fragments [37,38]. 

 Increasing internode length increased biomass of the surviving *M. micrantha* plants that emerged from the 0- and 2-cm burial depths, but burial had little effect on biomass. Burial in soils may significantly change the abiotic and biotic growth conditions such as temperature [39], photosynthetically active radiation [40], moisture [41], soil organic matter [41], rhizosphere oxygen content [6] and activity of soil organisms [42]. The overall results may depend on the balance between the stimulating and inhibiting effects of burial on plant growth [26], and contrasting results emerged from previous studies [15,43-46]. In the present study, burial delayed the time of emergence and thus decreased the time period for growth, but the surviving *M. micrantha* plants could recover gradually from burial. These results suggest that there may be some stimulating effects of burial on fragment growth, or the fragments survived from burial had high vigor compared with those that died. These explanations and forces together may explain the growth irresponsiveness of the species to burial. The result that increasing internode length increased the growth of the surviving *M. micrantha* plants is also consistent with previous findings [13,15,35,36] and suggests that reserves stored in stolon internodes can be remobilized and reused to increase plant growth [37,38].

 Burial at 2 cm slightly decreased shoot height and did not affect the root to shoot ratio, while increasing internode length increased shoot height and decreased the root to shoot ratio of the surviving *M. micrantha* plants. The higher root to shoot ratios in plants with a 0-cm internode length (Figure 3A) may be because of their low total biomass (Figure 2A). A number of studies reported evidence of shifts in resources from below to above ground plant parts [15,27,40], whereas other studies failed to detect it [43,47] or indicated that shifts were only possible at low or moderate burial levels [48]. In addition, root to shoot ratio may not change, but specific shoot length may increase with burial severity [47]. In the present study, light levels were reduced to simulate conditions in forest understory, and all plants may have increased biomass allocation to aboveground parts to increase emergence probability and light capture ability. However, it is also possible that the irresponsiveness of the root to shoot ratio to burial depth may partly explain the sensitivity of *M. micrantha* plant emergence to burial. 

 Overall, when buried at a 0-cm depth (i.e., at the soil surface), *M. micrantha* fragments exhibited a rather high resprouting rate compared with other stoloniferous invasive plants in China [16]. Burial at a 5-cm depth cannot prevent some fragments to emerge, and even burial at 8 cm cannot completely inhibit the emergence of clonal fragments attached with a long internode length (indicating a large storage of resources). In frequently disturbed habitats, storage of resources in stolon internodes may be an adaptive strategy because it increases regeneration capacity and growth of stoloniferous plants after fragmentation and burial. This may be one factor promoting the establishment, regeneration and invasiveness of *M. micrantha* populations in frequently disturbed habitats in South China. While plant fragmentation and movement of fragments and soil after disturbance may contribute to the invasion and spread of *M. micrantha*, for managing this species, it means that any processes (e.g., mechanical and manual control) producing stolon fragments and subsequent dispersal should be avoided. 
